# SARS and Pregnancy: A Case Report

**DOI:** 10.3201/eid1002.030736

**Published:** 2004-02

**Authors:** Corwin A. Robertson, Sara A. Lowther, Thomas Birch, Christina Tan, Faye Sorhage, Lauren Stockman, L. Clifford McDonald, Jairam R. Lingappa, Eddy Bresnitz

**Affiliations:** * Centers for Disease Control and Prevention, Atlanta, Georgia, USA; † New Jersey Department of Health and Senior Services, Trenton, New Jersey, USA; ‡ McKing Consulting, Atlanta, Georgia, USA; § Holy Name Hospital, Teaneck, New Jersey, USA

**Keywords:** severe acute respiratory syndrome, SARS, pregnancy, coronavirus, pneumonia, virus shedding, placenta previa, gestational diabetes, breast milk, breast feeding

## Abstract

We report a laboratory-confirmed case of severe acute respiratory syndrome (SARS) in a pregnant woman. Although the patient had respiratory failure, a healthy infant was subsequently delivered, and the mother is now well. There was no evidence of viral shedding at delivery. Antibodies to SARS virus were detected in cord blood and breast milk.

Severe acute respiratory syndrome (SARS) is a potentially life-threatening, atypical pneumonia that results from infection with a novel virus, SARS-associated coronavirus (SARS-CoV) (*1*–*3*). Limited studies and case reports suggest that other viral illnesses during pregnancy are sometimes associated with an increased risk for maternal illness and death (e.g., influenza) (*4*) and congenital anomalies (rubella and varicella) (*5*,*6*). No data exist regarding the effects of previously identified human coronaviruses on pregnancy. However, porcine reproductive and respiratory syndrome virus, an animal virus related to coronaviruses, is commonly associated with early fetal demise in pigs (*7*). In contrast, infection with feline infectious peritonitis virus, an animal coronavirus, results in newborn kittens’ becoming immune carriers of the virus (*8*). Data are limited regarding the effect of SARS-CoV on human pregnancy (*9*). We report additional details on the clinical course and outcomes of a case of laboratory-confirmed SARS-CoV infection in a pregnant woman (*10*).

## Case Report

A 36-year-old, previously healthy, Asian woman (gravida 2, para 1) at 19 weeks’ gestation with a low-lying placenta traveled in late February from the United States to Hong Kong with her husband and child. Before departing from the United States, the patient had been complaining of a mild, intermittent cough without fever for approximately 10 days. The cough, similar to one she had during her previous pregnancy, did not impair her ability to function. While in Hong Kong, between February 19 and March 2, 2003, she stayed at the same hotel and on the same floor as a physician from southern China, who is believed to have been the source of infection for patients who were the index case-patients in subsequent outbreaks of SARS in Hong Kong, Singapore, Hanoi, and Toronto, Canada (*11*). On February 24, fever, headache, weakness, anorexia, increasing cough, and shortness of breath developed in the patient. The next morning, she sought medical attention and was prescribed chlorpheniramine and acetaminophen. Her symptoms worsened, prompting her to see another physician 2 days later. A fetal ultrasound performed at this time was reportedly normal. Cephalexin was added to her regimen, but her condition did not improve; that night, she noted blood-tinged sputum.

On March 2, the patient returned to the United States where, acutely short of breath, she was hospitalized with pneumonia. Her highest temperature on admission was 102.5°F (39.2°C). Although chest auscultation was normal, chest radiography showed diffuse bilateral lower lobe infiltrates (Figure, part a). Admission arterial blood gas analysis showed pH 7.47, PaCO_2_ 31 mm Hg, and PaO_2_ 75 mm Hg on room air. Other pertinent laboratory findings included a leukocyte count of 3,300/mm^3^ (normal range 4,500–11,500/mm^3^) with a differential of 83% polymorphonuclear cells, 12% lymphocytes, and 5% monocytes; platelets of 103,000/mm^3^ (normal range 150,000–450,000/mm^3^); and alanine aminotransferase of 42 U/L (normal range 10–40 U/L). She was given supplemental oxygen for hypoxia and intravenous azithromycin and ampicillin to treat typical and atypical respiratory pathogens associated with community-acquired pneumonia. A fetal ultrasound performed on March 3 demonstrated a live intrauterine fetus of approximately 21 weeks gestational age and complete placenta previa. Despite antibiotic therapy, over the next 3 days, the patient became increasingly dyspneic; rales and decreased breath sounds developed, and she had radiographic evidence of progressive pulmonary infiltrates (Figure, part b). During this time, ticarcillin-clavulanate was added to her antimicrobial regimen, and rifampin was initiated as adjuvant therapy for possible legionellosis. Because the patient’s diagnosis remained elusive, tuberculosis was considered, and she was placed in airborne isolation. Arterial blood gas analysis on March 5 showed: pH 7.48, PaCO_2_ 31 mm Hg, and PaO_2_ 57 mm Hg on a 100% nonrebreather mask. The patient was subsequently placed on a mechanical ventilator. When avian (H5N1) influenza was considered in the differential diagnosis, oseltamivir was added to her therapy. During the next several days, she began to improve. Chest auscultation demonstrated few bibasilar rales, and a chest radiograph showed interval improvement (Figure, part c). She was afebrile by March 9 and extubated on March 12. On March 13, she had a fetal ultrasound that showed fetal growth consistent with dates and complete placenta previa. On March 17, she was discharged to home. Sputum, blood, and urine cultures; smears for acid-fast bacilli; and tests for *Legionella* urinary antigen, influenza nasopharyngeal antigen, and cold agglutinins were negative. Serum specimens collected 12 and 29 days after illness onset were tested at the Centers for Disease Control and Prevention (CDC) and found to be positive for SARS-CoV antibody.

**Figure:**
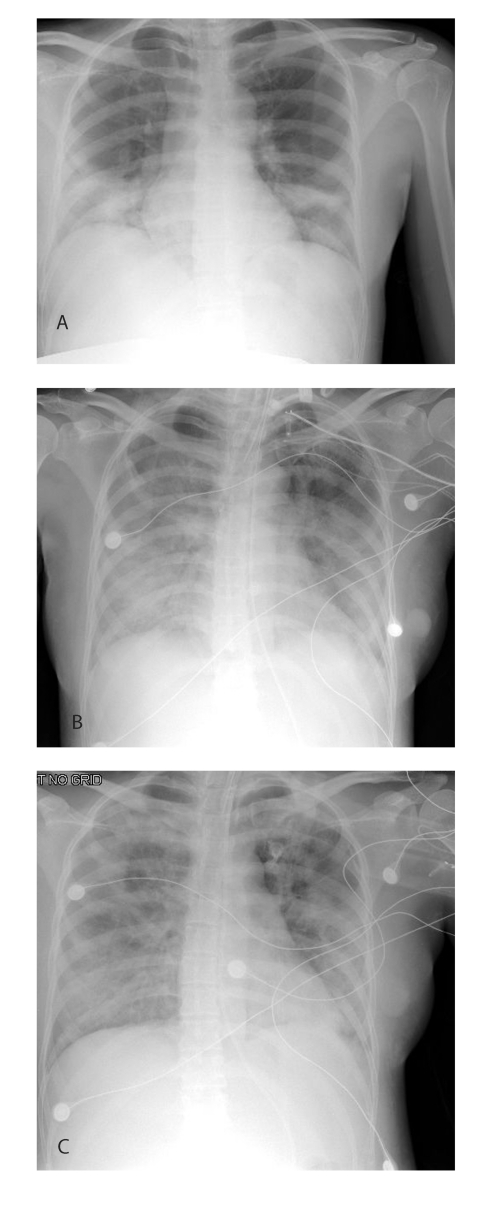
Chest radiographs of case-patient with severe acute respiratory syndrome (SARS) while pregnant. a, day 6 of illness; b, day 10; c, day 13.

A follow-up ultrasound examination on April 29 during routine prenatal care showed fetal growth consistent with dates and persistent complete previa. On May 2 (approximately 30 weeks’ gestation), the patient was diagnosed with gestational diabetes after an abnormal oral glucose tolerance test. Her diabetes was well-controlled by diet during the remainder of her pregnancy. Because serial ultrasounds performed on May 28 and June 24 demonstrated complete placenta previa, she underwent a cesarean section at 38 weeks’ gestation. A 6-lb, 15-oz (3,145-g) healthy female infant was delivered without complications. Apgar scores at 1 and 5 minutes were 9 and 9. Gross and microscopic inspection of the placenta did not show major abnormalities.

After informed consent was obtained, the following specimens (collected approximately 130 days after illness onset) were submitted to CDC for coronavirus testing: serum, whole blood, nasopharyngeal and rectal swab specimens from the mother, postdelivery placenta, cord blood, amniotic fluid, and breast milk. No viral RNA was detected in specimens tested by reverse transcriptase–polymerase chain reaction. Antibodies to SARS-CoV were detected in maternal serum, cord blood, and breast milk by enzyme immunoassay and indirect immunofluorescence assay (Table).

**Table:** Results of SARS-associated coronavirus (SARS-CoV) testing of specimens from case-patient infected while pregnant^a,b^

Specimen	SARS-CoV serologic results^c^	SARS-CoV RT-PCR
Maternal serum	+	ND
Maternal whole blood	–	ND
Maternal nasopharyngeal swab	ND	–
Maternal rectal swab	ND	–
Postdelivery placenta ^d^	–	–
Amniotic fluid	–	–
Cord blood	+	ND
Breast mlk	+	–

^a^SARS, severe acute respiratory syndrome; RT-PCR, reverse transcriptase–polymerase chain reaction; ND, not done. ^b^All specimens listed were collected 127 days after onset of the case-patient’s illness with the exception of breast milk, which was collected 131 days after illness onset. ^c^Serologic analysis included enzyme immunoassay and indirect immunofluorescence assay. ^d^Postdelivery placenta also underwent immunohistochemical (IHC) staining; there was no IHC evidence of SARS-CoV.

## Conclusions

On the basis of previous reports from Hong Kong, SARS infection can be associated with critical maternal illness, spontaneous abortion, or maternal death (*9*,*12*). We have described a serious SARS-associated illness that necessitated mechanical ventilation in a pregnant case-patient. Her pregnancy was also complicated by placenta previa and gestational diabetes—two conditions that she was at increased risk of developing because of advanced maternal age (*13*). The infant appeared unaffected by the mother’s SARS. However, at the time of delivery, clinical specimens from the infant were not available for SARS-CoV testing.

All healthcare workers involved in the delivery and subsequent care of the infant have remained healthy. However, serologic testing for SARS-CoV infection was not performed on these persons. The infant was delivered by cesarean section with contact, droplet, and airborne precautions in place (i.e., staff wore fit-tested N95 respirators and the cesarean section took place in a negative-pressure operating room). Since SARS-CoV was not detected in specimens collected at delivery and the patient delivered months after her illness onset, it is not clear if such precautions were necessary. However, other patients have demonstrated viral shedding in feces (*14*,*15*) and peritoneal fluid (*16*), suggesting that SARS-CoV may be present in other body fluids and hence, transmission during vaginal and cesarean deliveries is plausible. The presence, in this case, of SARS-CoV antibodies in cord blood and breast milk raises the issue of whether SARS-CoV infection during pregnancy results in passive immunity. Serial serologic testing of newborn clinical specimens and breast milk may provide a better understanding of the natural history of the fetal and newborn immune response to SARS-CoV infection during pregnancy.

This report, in conjunction with the reports from Hong Kong (*9*,*12*), provides an initial view of the spectrum of illness and outcomes associated with pregnancy-related SARS-CoV infection. A variety of factors might contribute to this range of outcomes (e.g., timing of SARS-CoV exposure during pregnancy; use of steroids, ribavirin, or both; differences in host immune response; the presence of coexisting conditions). More comprehensive epidemiologic and clinical summaries about the course of other SARS-affected pregnancies and long-term follow-up of infants are needed to fully define the pregnancy-related risks of this infection. Data on larger numbers of pregnant women infected with SARS-CoV may help refine infection-control strategies and provide a sound basis for clinical guidelines to manage future cases.
